# A model model: a commentary on DiFrancesco and Noble (1985) ‘A model of cardiac electrical activity incorporating ionic pumps and concentration changes’

**DOI:** 10.1098/rstb.2014.0316

**Published:** 2015-04-19

**Authors:** Katharine Dibb, Andrew Trafford, Henggui Zhang, David Eisner

**Affiliations:** 1 Institute for Cardiovascular Sciences, University of Manchester, Manchester, UK; 2 Computational Biology, Biological Physics Group, School of Physics and Astronomy, University of Manchester, Manchester, UK

**Keywords:** heart, computer model, pacemaker, calcium

## Abstract

This paper summarizes the advances made by the DiFrancesco and Noble (DFN) model of cardiac cellular electrophysiology, which was published in *Philosophical Transactions B* in 1985. This model was developed at a time when the introduction of new techniques and provision of experimental data had resulted in an explosion of knowledge about the cellular and biophysical properties of the heart. It advanced the cardiac modelling field from a period when computer models considered only the voltage-dependent channels in the surface membrane. In particular, it included a consideration of changes of both intra- and extracellular ionic concentrations. In this paper, we summarize the most important contributions of the DiFrancesco and Noble paper. We also describe how computer modelling has developed subsequently with the extension from the single cell to the whole heart as well as its use in understanding disease and predicting the effects of pharmaceutical interventions. This commentary was written to celebrate the 350th anniversary of the journal *Philosophical Transactions of the Royal Society*.

## 1. Introduction

It is almost exactly 30 years since Dario DiFrancesco and Denis Noble published their seminal paper modelling the electrical activity of cardiac muscle [1]. This was by no means the first paper to model such electrical activity. Indeed one of the authors (Noble) had much earlier, in 1962, produced the first mathematical model to reproduce the basic electrical properties of cardiac tissue [2]. This had been followed by various other models [3,4]. A PubMed search for ‘mathematical model heart action potential’ shows that in the first five years following the Noble 1962 model there were an average of two papers a year. This had increased to 178 per year in the period from 2009 to 2013. An obvious question is: what is special about the DiFrancesco and Noble paper to pay attention to it now? Our view is that it marks the transition from simply modelling the behaviour of surface membrane channels to considering the whole biology of the cell. The majority of previous models had considered the behaviour of surface membrane channels. They had, however, paid less attention to changes of intracellular ionic concentrations. The time leading up to the publication of the model had been a fertile one leading to many experimental observations which were ripe for embedding in a model. In the remainder of this paper, we consider some of the major ones and, in addition, how the field has moved on from the stimulus of this paper.

## 2. The pacemaker current

In the normal healthy heart, spontaneous beating is only observed in the sinoatrial node. Other sites, such as the atrioventricular node and His-Purkinje system, have the capacity to produce spontaneous activity but their intrinsic rate is slower than that of the sinoatrial node. Spontaneous beating results from a slow depolarization of the resting potential (pacemaker potential) leading to generation of an action potential. At the time of the paper, the underlying pacemaker current had recently undergone a reinterpretation. It had been thought that the pacemaker depolarization resulted from the decrease of a potassium conductance known as i_K2_ [5]. DiFrancesco showed, by contrast [6], that it was caused by an increase of an inward current, carried largely by sodium ions. Unlike most known currents which activate on depolarization, this channel activated on hyperpolarization. This funny (peculiar rather than ha-ha) nature of this current led to it being termed i_f_. An interesting question is that of how a sodium current could have masqueraded as a potassium one? A major factor that had led to this conclusion was the voltage dependence of the current. As the membrane potential was initially stepped more negative, following the initial jump, the current trace became more inward/less outward with time. As the magnitude of the hyperpolarization was increased, the time-dependent change of current reversed. These results were consistent with a decreasing potassium current which would be expected to reverse at a potential given by the Nernst potential of K^+^. However, as shown by DiFrancesco *et al*. [6,7] and modelled in fig. 17 of [1], a similar apparent reversal potential can result from the combination of (i) an increasing inward current and (ii) potassium entering the cell leading to a decrease in extracellular potassium concentration which would increase the outward current.

Subsequent work has led to the realization that pacemaker activity in the heart is even more complicated. It is now known that calcium is released from the sarcoplasmic reticulum (SR) during the pacemaker depolarization [8,9] and that this activates the electrogenic Na–Ca exchange (NCX) contributing to the depolarization. This calcium release component of pacemaker activity has been termed the ‘calcium clock’ and has been extensively modelled [10]. There is still considerable discussion about the relative contributions of this and the funny current to pacemaker activity [11]. The subject is of clinical interest as it underlies the development of drugs such as ivabradine which are designed to inhibit i_f_ and thereby slow the heart [12].

## 3. Extracellular potassium

Not long before publication of the paper [1], movement of potassium across the surface membrane had been shown to result in appreciable changes of potassium concentration in the restricted extracellular spaces of isolated cardiac tissue [13]. Considerable attention had been paid to the effect that these concentration changes would have on membrane currents [14–16]. As mentioned above, this problem had resulted in confusion of the identity of the pacemaker current. The fact that, at least in isolated tissues, extracellular potassium concentration can be different from that in the bulk bathing solution also affects the analysis of activation of the Na–K pump by external potassium ions [1,17]. The paper explicitly models changes of extracellular potassium. What is still unclear, however, is the extent to which such changes of extracellular K^+^ concentration occur in the normal, blood-perfused heart.

## 4. Intracellular sodium and the sodium pump

The first continuous measurements of intracellular sodium concentration (using sodium-sensitive microelectrodes) were published only seven years before DiFrancesco and Noble's paper [18]. Three years later, the activity of the sodium pump was directly measured from the electrogenic pump current it produces [17,19,20]. This experimental work was incorporated into the DFN model making it possible to consider the effects of changes of sodium pump activity. At least two aspects of sodium regulation were not apparent at the time this paper was written and could not therefore be included in the model. (i) As well as the voltage-dependent Na current and NCX, there is a significant contribution to Na^+^ entry from sodium hydrogen exchange [21,22]. (ii) There is also good evidence that the cardiac cell expresses more than one isoform of the sodium pump and the different isoforms are expressed in different cellular compartments (for review, see [23]). (iii) More recent work has also shown that the properties of the inward sodium current are different from what was known at the time of the paper. In particular, following the inactivation of the current, there is a persistent, non-inactivating component. DiFrancesco and Noble modelled one component of this current, the so-called ‘window’ current which results from the fact that over a narrow voltage range, the activation and inactivation curves overlap [24]. It is now known, however, that there is a further persistent Na^+^ current which is increased by hypoxia [25] and is a target for therapeutic intervention [26].

## 5. Intracellular calcium

As in many of its other aspects, mentioned above, the paper was a turning point in the incorporation of Ca signalling into electrophysiological models. The first measurements of changes of intracellular Ca during the heart beat had been obtained in frog cardiac muscle in 1978 [27]. Measurements in mammalian cardiac muscle appeared two years later [28]. The available data provided some estimate of the levels of systolic Ca but were not sensitive enough to record the lower, diastolic levels. Calcium release from the SR through the ryanodine receptor (RyR) was modelled in terms of calcium-induced calcium release [29], where Ca entering the cell triggered the release of much more from the SR. DiFrancesco and Noble were careful to point out, ‘We should emphasise that this part of the modelling is not thought to be too secure. There are too many arbitrary factors … ’ (p. 372). Unknown at the time, a major revolution in understanding of calcium release from the SR would take place only a few years later. A major concern with models of calcium-induced calcium release was how the release could be graded as opposed to being ‘all or none’. The problem was that release is initiated by calcium entering the cell via the L-type Ca channels. However, the calcium that is released from the SR will also increase intracellular Ca concentration ([Ca^2+^]_i_) and trigger further release. One would expect that such a process would result in the complete emptying of the SR. By contrast, it was already known that contraction and presumably therefore [Ca^2+^]_i_ was a continuous function of Ca entry [30]. Related to this, under normal conditions a local rise of [Ca^2+^]_i_ does not propagate along the cell. Only under conditions of elevated cell and SR Ca is such propagation observed [31]. Again, this suggested that something limits the strength of calcium-induced calcium release. This paradox was eventually resolved by Stern who demonstrated theoretically that graded release could result if the SR was controlled only by Ca channels in its immediate vicinity [32] (so-called ‘local control theory’). The experimental verification of this hypothesis was provided a year later with the observation of the Ca ‘spark’ representing release from a small cluster of SR Ca release channels [33]. Subsequent work showed that a major means for regulating the size of the Ca transient was by recruiting more and more of these release sites [34]. Models which incorporate this local release are much more complicated as the stochastic behaviour of every ion channel has to be allowed for.

Another, interesting aspect of modelling Ca regulation is the question of the various mechanisms by which Ca^2+^ enters the cell. At a normal resting potential, essentially all the L-type Ca channels are closed. Ca^2+^ is still being pumped out of the cell, however, by NCX. This raises the question of the route by which Ca^2+^ enters to balance this efflux. DiFrancesco and Noble assigned a background, leak influx in their model. A later, experimental paper found that when the L-type channel and NCX are inhibited, hyperpolarization indeed increases [Ca^2+^]_i_ in a manner expected for such a leak [35]. An unanswered question is the molecular basis of this leak. Whatever its origin, it may well not be important in the beating heart where the regular influx of Ca into the cell on the L-type current will presumably dominate over this entry.

## 6. Sodium–calcium exchange

At the time that the paper appeared, a revolution had recently occurred in understanding the importance of NCX. NCX uses the energy provided by Na^+^ ions entering the cell down their electrochemical gradient to pump Ca^2+^ out of the cell. For several years after its identification in the heart and squid axon [36,37], it was unclear how many Na^+^ exchanged for each Ca^2+^. It was realized that an electroneutral NCX (2 Na^+^ per Ca^2+^) could not provide enough energy to account for the low resting [Ca^2+^]_i_. A value of 3 or more Na^+^ would explain the resting [Ca^2+^]_i_ but would mean that the NCX generated an electrical current. This idea was championed by Lorin Mullins, who also suggested that the resulting current could account for aspects of electrical behaviour of the heart [38,39], which was then put into a cardiac model [40]. There was, however, a lack of direct evidence showing this experimentally [41]. Indeed the direct demonstration of an electrogenic NCX current in the heart appeared only after DiFrancesco and Noble's paper was published [42,43]. DiFrancesco and Noble assumed a value of 3 Na^+^ which correctly anticipated the subsequent experimental finding.

## 7. Other currents

Several membrane currents which contribute to cardiac electrophysiology were unknown at the time of writing the paper. These include the ATP-sensitive K current [44]. The division of the Ca current into L- and T-type [45] was also unknown. The DFN model includes the transient outward current which underlies the initial phase of repolarization. This was modelled as a Ca-activated potassium current. More recent work has shown the complexity of this transient outward current. It comprises a voltage-activated K current [46] and, in some species, there is a contribution from a Ca-activated chloride current [47]. There is evidence for the existence of Ca-activated K currents, particularly in heart failure [48,49], but their precise functional role still remains somewhat unclear. It may seem surprising that the model does such a good job of reproducing the properties of the action potential. This highlights a challenge for computer modelling as the ability to reproduce the action potential is no guarantee that the correct mechanisms are included.

## 8. Spatial localization

The DFN model treated the cytoplasm as a homogeneous structure and also regarded the surface membrane as a single structure. Including cytoplasmic ion changes was a significant advance, but an enormous amount of subsequent work has shown that, for the following reasons, these were substantial simplifications. Transverse (t) tubules make up much of the surface membrane of the cell, and there is evidence that their composition of membrane proteins differs from that of the rest of the surface membrane (for review, see [50]). It is also clear that the space between the transverse tubule and the SR (the so-called ‘dyadic space’) has a very small volume. Consequently fluxes of, for example, sodium or calcium into the cell will result in much larger changes of concentration than would be the case if the ions distributed equally throughout the cytoplasm [51]. More recent models incorporate detailed ultrastructural information (e.g. [52]).

## 9. Computer models of subcellular signalling

The cellular events modelled by DiFrancesco and Noble do not act in isolation but are regulated by a complex array of intracellular signalling pathways. The complexity and interdependence of known cellular signalling networks is such that computer modelling can serve a useful role in (i) identifying potential mechanisms of normal function, (ii) probing how cell signalling can contribute to diseases like heart failure and (iii) identifying which pathways may be useful for drug development. For example, in recent years the β-adrenergic signalling cascade and Ca-calmodulin-dependent protein kinase II (CAMKII) cellular signalling have been implicated in disease and arrhythmogenic mechanisms. This has necessitated the development of a large number of computer models based on signalling pathways to investigate causative mechanisms. Such simulations have yielded information which can be difficult to obtain experimentally (reviewed in [53,54]). CAMKII was first incorporated into a model of canine excitation contraction coupling to understand its role in the rate dependence of Ca^2+^ handling [55]. This study suggested CAMKII to be important in the rate dependence of the Ca^2+^ transient but not the action potential duration. Modelling of the effect of CAMKII on Na^+^, Ca^2+^ and K^+^ currents suggests that, while individually opposing effects on action potential duration were observed (whereby effects on *I*_Na_ and *I*_Ca_ tended to prolong action potential duration but CAMKII *I*_to_ modulation shortened the action potential), combining the effects on all three channels resulted in action potential shortening, highlighting how computer modelling is able to separate individual effects of signalling molecules [56]. More recently, a computer model has been used to explore the relationship between RyR and CAMKII; this model describes how individual L-type Ca^2+^ channels and RyRs may respond to local CAMKII and dyadic Ca^2+^ [57], which would be very difficult to measure experimentally. Thus expansion of computer modelling to encompass cardiac signalling networks now aids our understanding of the underlying components of the cellular behaviours modelled by DiFrancesco and Noble.

## 10. Towards the whole heart

DiFrancesco and Noble modelled a single cell or Purkinje fibre. They left to future work the spread of excitation from one cell to another in a real heart consisting of billions of cells. Indeed, it would have been very difficult, if not impossible, for the computer power of the 1980s to easily allow for this. It has become increasingly clear that many important features depend on this multicellular nature, including the intrinsic electrical heterogeneity of cardiac tissue and anisotropic cell-to-cell electrical coupling arising from the arrangement of cardiac tissue fibres [58,59]. Whole heart modelling was made possible by the seminal work of Nielsen *et al.* [60], who reconstructed the first three-dimensional realistic computer model for the anatomic structures of cardiac tissue. Such three-dimensional anatomical models allow integration of cardiac electrophysiology with anatomical structures. However, until the early 1990s, owing to the limitation of computing power and other challenges [61], large-scale computer modelling of cardiac tissues used grossly simplified representations of cardiac electrophysiology with idealized cardiac tissue geometry [62], or biophysically detailed cardiac electrophysiology with idealized cardiac tissue geometry [63]. It then evolved into whole heart modelling with implementations of three-dimensional anatomical structures but still with simplified models of cellular electrophysiology [64]. With the rapid advance of high-performance computing and visualization techniques in the last decade, a virtual heart model that integrates both detailed electrophysiology and anatomical structures became possible [65].

**Figure 1.**
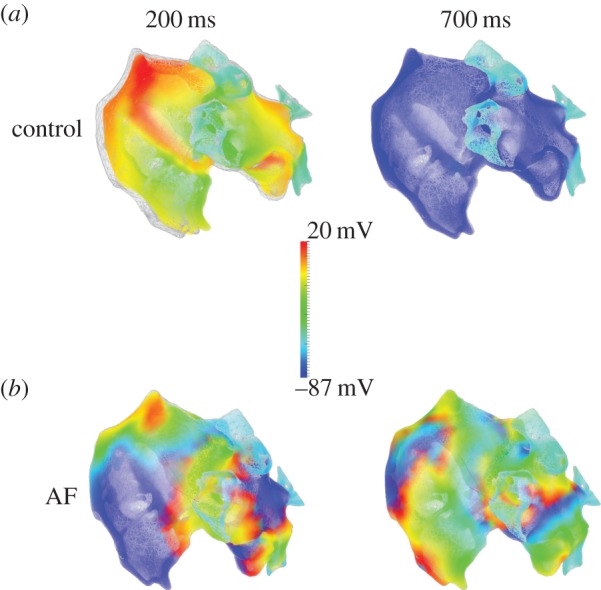
Simulated electrical and mechanical activities of the human atria during control and chronic atrial fibrillation conditions. Atrial electrical excitation waves are presented by colour coded cellular action potentials (see colour key), and mechanical contraction is represented by superimposition of the atrial mesh on its original geometry (grey; i.e. the geometry before electrical activation). (*a*) Snapshots of atrial electromechanical activity at 200 ms (during contraction) and 700 ms (after repolarization and tissue relaxation). (*b*) During atrial fibrillation (AF), showing negligible contraction and multiple re-entrant wavelets which are maintained and persist at 700 ms. (Figure was produced by Dr Ismail Adeniran and H.Z.)

Mathematically, cardiac tissue can be idealized as a spatially extended syncytium consisting of billions of electrically coupled cells that are bounded by the anatomical geometry of the heart. It should be remembered that, as well as myocytes, the heart also contains fibroblasts which are electrically coupled to the myocytes [66]. The spread of excitation can be represented by a reaction–diffusion equation, in which the electrical activities of individual cells are represented by single-cell models and the intercellular couplings are modelled via diffusive interactions of membrane potentials by a gap junctional conductance [59]. Within this mathematical frame, models of the whole heart incorporating detailed anatomical structures and electrophysiology have been developed for various species and different regions of the heart [67–71]. An example is shown in figure 1. A clear example of applications of these large-scale models of the propagation of the action potential through the heart is to investigate how the propagation can breakdown, resulting in the genesis of re-entrant excitation waves that are related to cardiac arrhythmias in normal [72] and gene mutation conditions [73].

Pumping blood around the body requires a sequence of rhythmic mechanical contractions from the heart; these are triggered by the propagation of excitation waves. The conduction of excitation waves in the heart generates an electrical field in the surface of the body, which can be measured as the body surface ECGs. Current development of computing power also allows development of multi-scale electromechanical models of the heart, which integrate coupling among cardiac electrophysiology, cellular contraction mechanisms and mechanic deformation [74,75]. The whole heart model can be embedded into the torso model, enabling simulations of the body surface potential and ECGs in normal and pathological conditions [76].

## 11. Broader perspectives

In the future, it is hoped that computer models will not only help us to understand mechanisms of cardiac function in health and disease, but actually guide therapy to aid in the treatment of disease. The vision for the Virtual Physiological Human initiative is to generate a customized computer model of a patient's condition across multiple organ systems by creating an infrastructure to link models at different biological levels to allow prediction of personalized medication [77]. Current work, however, has already made important steps using computer models to facilitate patient treatment. At the organ level, compared to other tissues, the computational model of the heart is one of the most highly advanced [78]. This has been facilitated by the long history of cardiac modelling and seminal contributions such as that of DiFrancesco and Noble. Models of the whole heart are being used to investigate how resynchronization therapy may be improved in dyssyncronous heart failure [79]. Advanced imaging methods make the construction of a patient-specific model possible. Such a personalized model has been used to simulate ventricular tachycardia (VT), opening up the possibility of using computer modelling to assess the risk of VT in a given patient and also plan potential ablation strategies for treatment [80]. The ability of computer models to screen for drugs which may be arrhythmogenic has also been demonstrated. While reduction of *I*_Na_ in the Cardiac Arrhythmia Suppression Trial (CAST) [81] was expected to decrease arrhythmias, sudden cardiac death actually increased, an effect which was not predicted by conventional pharmacological studies [82]. A computer model of single cells showed that both lidocaine and flecainide lowered excitability but cells were still able to generate action potentials. When the model was scaled up to incorporate coupled groups of cells it showed that flecainide caused serious conduction block at high heart rates which was confirmed experimentally in rabbit heart. The model was also able to successfully predict concentrations of both drugs at which adverse effects were seen, which was again validated experimentally in the rabbit heart [83]. This type of model may therefore be able to highlight drugs which exacerbate arrhythmias in the future. As with all computer models, it is important to stress that data should be validated using experiments which are separate from those used to define assumptions within the model itself. Computer models will never completely replace experiments on tissues and animals.

## 12. Summary

Looking back 30 years, the DiFrancesco and Noble paper still appears as a superb mathematical model of cardiac electrophysiology. Equally importantly, it can be seen as a vital bridge in the development of mathematical models of the heart which took the field from simple biophysics to systems biology. In particular, its consideration of changes of ionic concentrations can be seen as the forerunner of countless subsequent models.

## Author profiles

**Katharine Dibb** received her PhD from Leeds University and is currently a lecturer at the University of Manchester.

**Andrew Trafford** is a British Heart Foundation Senior Fellow and Professor of Cardiac Pathophysiology at the University of Manchester. He qualified as a veterinary surgeon and obtained his PhD at the University of Liverpool.

**Henggui Zhang** received his PhD from Leeds University and is currently Professor of Biological Physics at the University of Manchester.

**David Eisner** is currently British Heart Foundation (BHF) Professor of Cardiac Physiology at the University of Manchester. From 1976 to 1979, he studied for his D. Phil in Denis Noble's laboratory in Oxford at the same time that Dario DiFrancesco was a visiting scientist. He can still remember the excitement when Dario first revolutionized cardiac pacemaker activity.
